# Impact of alcohol disorder and the use of illicit drugs on tuberculosis treatment outcomes: a retrospective cohort study

**DOI:** 10.1186/s13690-018-0287-z

**Published:** 2018-07-12

**Authors:** Daniele M. Pelissari, Fredi A. Diaz-Quijano

**Affiliations:** Department of Epidemiology, School of Public Health, University of São Paulo, Av. Dr. Arnaldo, 715, São Paulo, 01246-904 Brazil

**Keywords:** Tuberculosis, Illicit drugs use, Alcohol disorder, Outcome, Interaction

## Abstract

**Background:**

Alcohol and illicit drugs are associated with the discontinuation of tuberculosis (TB) treatment and can compromise the immune system. We estimated the impact of alcohol disorder and the use of illicit drug on TB treatment outcomes, considering the interaction of both substances in patients from São Paulo state, Brazil.

**Methods:**

This is a retrospective cohort of patients diagnosed with TB from 2011 to 2015. We estimated the relative risk (RR) of an unsuccessful outcome associated with alcohol disorder, use of illicit drugs and their interaction using a multiple regression model. We used the adjusted RR to estimate the population attributable fraction.

**Results:**

Out of a total 77,212 TB patients, 22.2% used at least one of the substances of interest during treatment, while 17% presented an unsuccessful outcome of TB treatment. Compared with no exposure to any substance, alcohol disorder alone (adjusted RR: 1.48; 95% CI: 1.4–1.56), drug use alone (adjusted RR: 2.1; 95% CI: 1.98–2.21) and exposure to both substances (adjusted RR: 2.09; 95% CI: 1.97–2.21) were all associated with a higher risk of an unsuccessful outcome. The adjusted RR of an unsuccessful outcome for people exposed to both substances was 32.7% (95% CI: 26.8–38.2%) and 15.8% (95% CI: 11.5–20.1%) lower than expected on the multiplicative and additive scales respectively. Among all TB patients, 15.8% (95% CI: 15–16.5%) of unsuccessful outcomes was attributable to those exposures.

**Conclusions:**

We identified a negative interaction between alcohol disorder and the use of illicit drugs on TB treatment outcomes. Despite this, interventions to reduce substance use in TB patients could have a meaningful contribution to preventing unsuccessful treatment outcomes.

**Electronic supplementary material:**

The online version of this article (10.1186/s13690-018-0287-z) contains supplementary material, which is available to authorized users.

## Background

In 2016, 10.4 million new tuberculosis (TB) patients and 1.67 million deaths from the disease were registered worldwide [1]. Brazil is a middle-income country in South America. It is included in the list of the highest TB and TB-HIV burden countries issued by the World Health Organization (WHO) [1]. In 2017, 69,569 new TB patients were registered in the country; only 73% of them were cured [2]. The high percentage of unsuccessful outcomes in Brazil perpetuates transmission of the disease at the community level. This finding compromises the country’s likelihood of meeting the target of 90% reduction in TB incidence and 95% reduction in TB deaths by 2035, as set by the WHO’s End TB Strategy [3].

Alcohol consumption is related to pharmacokinetic alterations of the medicines used in TB treatment [4], and the use of both alcohol and illicit drugs is associated with discontinuing treatment [5–7] and compromising the immune system [8]. Therefore, these substances have common mechanisms to affect TB treatment outcomes, possibly leading to either a saturation or an amplification of their effects.

None of the existing epidemiological studies that analysed the association of those substances with TB treatment outcomes have tested the interaction between both exposures [5–7, 9]. However, understanding their relationship could be essential to properly estimating the population impact of these potentially modifiable risk factors on unsuccessful TB treatment outcome. Consequently, we estimated the impact of alcohol disorder and the use of illicit drugs on TB treatment outcomes considering the interaction of both substances, in patients from São Paulo state, Brazil.

## Methods

This is a retrospective cohort of TB patients diagnosed from 2011 to 2015, based on surveillance data from the Tuberculosis Cases Notification and Monitoring System (TBWEB) updated in May 2017. This system only covers São Paulo state, representing approximately 25.5% of Brazilian new TB cases [2]. TBWEB records data collected by health professionals in a standardized TB notification form and allows follow-up on TB outcomes even if patients are transferred among health facilities. As a result, only a few cases in the study period did not have outcome data; when it did happen, it was mainly related to transfers out of São Paulo state.

### Study population

We included new TB cases (never treated before or those that previously had received TB treatment for a period ≤1 month), aged ≥15 years, and who started TB treatment during the study period. We intended to focus the study on drug-sensitive TB cases; therefore, we excluded those patients recorded as having TB drug-resistant. From the possible study population of 79,075 patients, 1863 (2.4%) patients did not have a TB outcome recorded. Therefore, in total, 77,212 patients were available for the outcome analysis.

As we included all patient information available during the study period, a sample size was not calculated in advance. Given the number of patients included, the frequency of exposures to substances (Table 1) and an incidence of the outcome in the unexposed group of 14.3%, this study had a power higher than 99% to evaluate associations with a relative risk (RR) of 1.2 or greater for any of the exposure categories (alcohol, drugs or both).

**Table 1:** Factors associated with unsuccessful tuberculosis treatment outcome, São Paulo-state, Brazil, 2011–2015

Characteristics	Total	Incidence of unsuccessful outcome of tuberculosis treatment		Crude RR (95% CI)^a^	Adjusted RR (95% CI)^a^
		No.	%		
Overall	77,212	13,300	17		
Alcohol and illicit drug					
Neither	60,107	8607	14.3	1	1
Only alcohol disorder	7487	1765	23.6	**1.65 (1.57–1.72)**	**1.48 (1.4–1.56)**
Only drug use	5199	1472	28.3	**1.98 (1.89–2.07)**	**2.1 (1.98–2.21)**
Alcohol disorder and drug use	4418	1456	33	**2.3 (2.2–2.41)**	**2.09 (1.97–2.21)**
Sex					
Female	22,498	3102	13.8	1	1
Male	54,714	10,198	18.6	**1.35 (1.3–1.4)**	**1.27 (1.22–1.32)**
Age (years)					
15–34	36,080	5370	14.9	1	1
34–49	21,681	4068	18.8	**1.26 (1.21–1.31)**	0.99 (0.94–1.03)
50 and more	19,451	3862	19.9	**1.33 (1.28–1.38)**	**1.23 (1.18–1.29)**
Race					
Non-black	35,707	5727	16	1	1
Black	33,135	5951	18	**1.12 (1.08–1.16)**	**1.13 (1.09–1.17)**
HIV					
No	69,973	10,582	15.1	1	1
Yes	7239	2718	37.5	**2.48 (2.4–2.57)**	**2.16 (2.07–2.26)**
Clinical form					
Pulmonary	65,271	11,364	17.4	1	1
Extrapulmonary	11,903	1899	16	**0.92 (0.88–0.96)**	**0.86 (0.82–0.9)**
Prison					
No	68,228	12,608	18.5	1	1
Yes	8984	692	7.7	**0.42 (0.39–0.45)**	**0.51 (0.47–0.56)**
Homeless					
No	75,218	12,278	16.3	1	1
Yes	1993	1022	51.3	**3.14 (3–3.29)**	**2 (1.89–2.13)**
Directly observed treatment					
No	16,791	4237	25.2	1	1
Yes	54,940	7198	13.1	**0.52 (0.5–0.54)**	**0.55 (0.53–0.57)**

*RR* relative risk, *95% CI* 95% confidence interval

^a^Boldface indicates statistical significance (*p* < 0.05)

### Variables

Using WHO definitions as adapted to TBWEB, the main TB treatment outcomes were classified as treatment success (cured or completed treatment) or unsuccessful TB treatment outcome (death and lost to follow-up) [10].

Alcohol disorder, the use of illicit drugs and an interaction between them were analysed as the main independent variables. Alcohol disorder is collected in the TB notification form as the presence or not of an associated disorder and is categorized as yes or no. For the definition of alcohol disorder, Brazil adopts the American Psychiatric Association definition [11]. The use of illicit drugs is also collected in the notification form as a dichotomous variable (yes or no).

Substance exposures were identified by health professionals during clinical interviews. Thus, substance exposures were routinely recorded at the time of diagnosis, which is before the start of treatment.

### Statistical analysis

We estimated the RRs and their 95% confidence intervals (95% CI) in a multiple model using Poisson regression with robust variance [12]. We obtained a model with the following structure:

$$ Ln(y)={\beta}_0+{\beta}_a{X}_a+{\beta}_d{X}_d+{\beta}_{a\wedge d}{X}_{a\wedge d}+\sum \limits_{i=1}^k{\beta}_i{C}_i $$

where *y* represents the predicted value of the dependent variable; *β*_0_ is the intercept; *β*_*a*_, *β*_*d*_ and *β*_*a* ∧ *d*_ represent the regression coefficients of the dichotomous independent variables (adopting values of 0 [no] or 1 [yes]) that correspond to exposure to alcohol (*X*_*a*_), to illicit drugs (*X*_*d*_) and to the interaction term defining the concomitant exposures to both alcohol and drugs (*X*_*a* ∧ *d*_) respectively.

This model was adjusted by a number (*k*) of covariates (*C*_*i*_) with their corresponding coefficients (*β*_*i*_). We considered the following covariates, which have been recognized as factors associated with TB outcome in previous studies [5–7, 9]: sex; age group (15 to 34; 35 to 49, 50 and older); race (blacks and non-blacks); HIV status; clinical form of TB (pulmonary [includes mixed form] and extrapulmonary); prisoner; homeless population; and directly observed treatment (DOT).

From this model, compared with the category of non-exposed to either alcohol or drug, the adjusted RRs for only alcohol (*RR*_*a*0_), only drug (*RR*_0*d*_) and for the exposure to both substances (*RR*_*ad*_) were calculated respectively as: *RR*_*a*0_ = exp(*β*_*a*_); *RR*_0*d*_ = exp(*β*_*d*_); and *RR*_*ad*_ = exp(*β*_*a*_ + *β*_*d*_ + *β*_*a* ∧ *d*_).

We calculated the ratio of RRs as a measure of interaction on the multiplicative scale, consisting of the ratio of *RR*_*ad*_ to the RR expected from the product of the effects of the two exposures considered separately [13]. Then,

$$ ratio\ of\  RRs=\frac{RR_{ad}}{RR_{a0}{RR}_{0d}}=\exp \left({\beta}_{a\wedge d}\right) $$

We also calculated the relative excess risk due to interaction (RERI) as a measure of additive interaction [13] as follows: *RERI* = *RR*_*ad*_ − (*RR*_*a*0_ + *RR*_0*d*_ − 1) = *RR*_*ad*_ − *RR*_*a*0_ − *RR*_0*d*_ + 1.

With a ratio of RRs > 1 and RERI> 0, we would consider an interaction as positive in the multiplicative and the additive scales, respectively. However, in the case of a ratio of RRs < 1 and a RERI< 0, we interpreted the interaction as negative and calculated how much lower the *RR*_*ad*_ was than expected in both scales. Thus, we defined that proportion lower than expected in the multiplicative scale as: $1-\frac{RR_{ad}}{\left({RR}_{a0}{RR}_{0d}\right)}$; and in the additive scale as: $1-\frac{RR_{ad}}{\left({RR}_{a0}+{RR}_{0d}-1\right)}$.

Subsequently, we simulated the incidence expected from the adjusted RR using the incidence observed in the population not exposed to either alcohol disorder or use of illicit drugs as a reference. These incidences were then compared with those expected in additive and multiplicative scales, based on the sum of attributable risk and the product of RRs respectively. We followed the recommendations made by Knol and VanderWeele [14] to present interaction analyses.

Finally, we estimated the population attributable fraction (PAF) [15] of the use of substances for the risk of unsuccessful outcome of TB treatment using the formula:

$$ \mathrm{PAF}={p}^{\hbox{'}}\frac{\theta -1}{\theta } $$

where p’ is the prevalence of exposure to substances in the non-successful treatment population, and θ is the adjusted RR estimated by the regression model. PAF and interaction measures derived from the regression model were calculated using a nonlinear combination of parameter estimates based on the delta method [16].

To evaluate whether missing outcomes could affect the results, we first compared profile patients with and without treatment outcome registration using the Pearson Chi-square test (Additional file 1: Table S1). Because those populations were slightly different, we ran a sensitivity analysis with all unrecorded treatment outcomes set to either successful or unsuccessful (Additional file 2: Table S2).

All analyses were performed on Stata 12 (Stata Corporation, Texas USA).

## Results

Of the total of 77,212 TB patients, 22.2% were exposed to at least one of the conditions (alcohol disorder or use of illicit drugs) at the beginning of TB treatment, and the overall incidence of unsuccessful outcome was 17%. In the multiple model, exposure to only alcohol disorder (RR: 1.48; 95% CI: 1.4–1.56), to only use of illicit drugs (RR: 2.1; 95% CI: 1.98–2.21) and to both substances (RR: 2.09; 95% CI: 1.97–2.21) were all associated with a higher risk of unsuccessful treatment outcome compared with patients not exposed to any of these conditions. The other risk factors significantly associated with a higher risk of the unsuccessful treatment outcome were: male sex, age over 50 years (compared with the group aged between 15 and 34 years), black race, coinfection with HIV and homeless population. Protective factors were extrapulmonary clinical form, prisoners and DOT (Table 1). All these associations were also observed in the models considered in the sensitivity analysis (Additional file 2: Table S2).

The adjusted RR for the group exposed to both alcohol disorder and illicit drug use (RR: 2.09; 95% CI: 1.97–2.21) was 32.7% (95% CI: 26.8–38.2%) lower than expected on the multiplicative scale and 15.8% (95% CI: 11.5–20.1%) lower than expected on the additive scale (*P* < 0.001 for interaction on both scales) (Table 2). Consequently, the ratio of RRs was 0.67 (95% CI: 0.62–0.73; *P* < 0.001) and the RERI was negative (− 0.49; 95%CI: -0.65 - -0.33; *P* < 0.001). Thus, we observed a negative interaction of alcohol disorder and drug use on the TB outcome that was statistically significant in both the multiplicative and additive scales.

**Table 2:** Interaction between alcohol disorder and illicit drug use on the risk of unsuccessful tuberculosis treatment outcome, São Paulo-state, Brazil, 2011–2015

	Non-illicit drug use		Illicit drug use		RRs (95% CI)^a^ for illicit drug within strata of alcohol
	No. of unsuccessful outcome/successful outcome	RR (95% CI)^a^	No. of unsuccessful outcome/successful outcome	RR (95% CI)^a^	
Non-alcohol disorder	8607/51500		1472/3727		
		1		2.1 (1.98–2.21) *P* < 0.001	2.1 (1.98–2.21) *P* < 0.001
Alcohol disorder	1765/5722		1456/2962		
		1.48 (1.4–1.56) *P* < 0.001		2.09 (1.97–2.21) *P* < 0.001	1.41 (1.31–1.51) *P* < 0.001
RRs (95% CI)^a^ for alcohol within strata of illicit drug		1.48 (1.4–1.56) *P* < 0.001		1.00 (0.93–1.06) *P* = 0.90	

*RR* relative risk, *95% CI* 95% confidence interval

^a^Adjusted for sex, age, race, HIV, clinical form of TB, prison, homeless and directly observed treatment

**Fig. 1:**
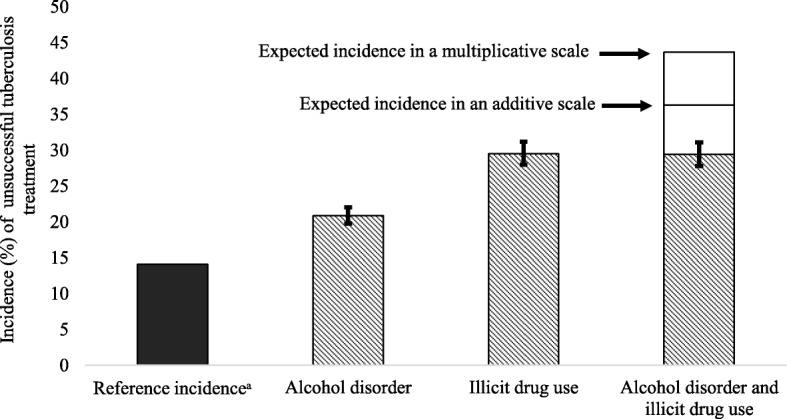
Incidence of unsuccessful tuberculosis treatment outcome predicted by adjusted relative risk vs. that expected in multiplicative and additive scales according to alcohol disorder or illicit drug use, São Paulo-state, Brazil, 2011–2015. ^a^Reference incidence corresponds to the incidence observed in patients not exposed to either alcohol or illicit drugs (14.3%). Other incidences presented correspond to the product of the reference incidence and the corresponding relative risk adjusted by sex, age, race, HIV, clinical form of TB, prison, homeless, and directly observed treatment

According to the simulation, the expected incidences of unsuccessful outcome in the group exposed to both substances would be 36.3 and 43.7% in the additive and the multiplicative scales, respectively. However, the incidence predicted by the adjusted RR of the group exposed to both substances was 29.4% (Fig. 1).

From the multiple model, the estimated PAFs for an unsuccessful outcome of treatment were 4.3% (95% CI: 3.8–4.8%), 5.8% (95% CI: 5.5–6.1%) and 5.7% (95% CI: 5.4–6.0%) for only alcohol disorder, only illicit drug use and both exposures, respectively. The joint PAF for these factors totalled 15.8% (95% CI: 15.0–16.5%). According to the sensitivity analysis, this joint PAF would be 15.8% (95% CI: 15.1–16.6%) if all patients with a missing outcome were set to successful treatment and 13.8% (95% CI: 13.1–14.5%) if they were set to unsuccessful treatment (Table 3).

**Table 3:** Adjusted fraction of unsuccessful outcomes in a population of TB patients that was attributable to alcohol disorder and illicit drug use (PAF%), São Paulo-state, Brazil, 2011–2015

Alcohol and illicit drug	Main analysis^a^ PAF (95% CI)	Model 1^a,b^ PAF (95% CI)	Model 2^a,b^ PAF (95% CI)
Neither			
Only alcohol disorder	4.3 (3.8–4.8)	4.3 (3.8–4.8)	3.7 (3.3–4.2)
Only drug use	5.8 (5.5–6.1)	5.8 (5.5–6.1)	5 (4.8–5.3)
Alcohol disorder and drug use	5.7 (5.4–6.0)	5.7 (5.4–6)	5 (4.7–5.3)
Total	15.8 (15.0–16.5)	15.8 (15.1–16.6)	13.8 (13.1–14.5)

*PAF* Population Attributable Fraction, *95% CI* 95% confidence interval

^a^Estimates based on relative risks adjusted by sex, age, race, HIV, clinical form of TB, prison, homeless and directly observed treatment

^b^Model 1- unobserved treatment outcomes set to successful; model 2- unobserved treatment outcomes set to unsuccessful

We represented the key messages of the association of alcohol disorder and illicit drug use on tuberculosis treatment outcome in an infographic in the Additional file 3: Figure S1.

## Discussion

Our study showed a statistically significant interaction between alcohol disorder and illicit drug use on unsuccessful TB treatment outcomes. Specifically, the incidence of unsuccessful outcomes among patients exposed to both alcohol disorder and illicit drug use was lower than that expected from either the sum or the product of the estimated effects of the two exposures considered separately.

According to our results, association with only the use of illicit drugs was similar to that with both substances; therefore, alcohol disorder did not seem to contribute with an additional risk to illicit drug users. Illicit drug use can influence TB outcomes through its biological effects, social marginalization or by affecting adherence to treatment, which are mechanisms also associated with alcohol addiction [5, 7, 8]. However, we hypothesize that the use of illicit drugs (more than alcohol disorder) has the potential to influence and transform a patient’s social network, creating obstacles to family support [17]. In addition, the illegal character of drug use, treatment barriers, including poor adherence and limited access to care, represent major challenges for treatment of drug users [18] than alcohol users.

In our study, we found that 14.5% of TB patients had alcohol disorder, and 12.7% used illicit drugs. The prevalence of alcohol disorder in the TB patients from this study was similar to that observed in the Brazilian capital’s population aged 8 years and older in 2013 (16.4%) [19]. Data about drug consumption are not routinely collected in Brazil. However, in 2005, a survey conducted with 200,000 Brazilians aged 12 or older estimated that the use of drugs (except alcohol and tobacco) in the past month was 4.5% [20], noticeably lower than that observed in the study’s population of TB patients. Because the general population may be less likely to admit illegal drug use, information bias could be a possible explanation for these differences. Alternatively, the higher risk of TB among illegal drug users [18] could explain the higher prevalence of drug use in TB patients compared with the general population.

We also found that even with the negative interaction, almost 16% of unsuccessful treatment outcomes were attributable to substance exposures. Eliminating those substances from the population would be difficult, but reducing consumption to a much lower level is possible. One of the most effective strategies, tested by Milkman in teenagers in Iceland, is replacing the sensation created by substance use for one provided by the practice of physical activities they wanted to do [21]. With this strategy, the rates of alcohol usage in the past month decreased from 42% in 1998 to 5% in 2016, and of cannabis from 17 to 5% [21]. Although challenging, government officials in Iceland turned that strategy into public policies, increasing public funding for youth sports and clubs, among other strategies [22].

### Limitations

Underreporting of exposures and non-standardized data collection procedures could lead to an information bias. However, we believe that any underreporting or misclassification of the exposures would be no differential between the categories of the outcome and therefore would lead to an underestimation of the associations. Another limitation is that the routine surveillance system used as the data source in this retrospective cohort does not contain detailed information about levels of consumption. This limitation could lead to an incomplete adjustment and limit the analysis of the interaction mechanism. Local legislation defines the list of the illicit substances [23]. In a 2015 survey, the illicit drugs most used in Brazil were reported to be cannabis (50.8%), lysergic acid diethylamide (LSD) (19.5%), cocaine (12.8%) and ecstasy (12.2%) [24]. However, information about the type of substance of illicit drug is not systematically collected so was not possible to be analysed in this study.

Despite these limitations, the exposure categories we used in this study are widely used by the surveillance system. Moreover, because substance exposures are recorded before starting the TB treatment, our findings support that alcohol disorder and use of illicit drugs are predictors of an unsuccessful outcome. Therefore, this study suggests that routine collected information about substance exposures can be useful to identify TB patients at risk of unsuccessful outcomes and focus on preventive strategies.

## Conclusion

We identified a negative interaction between alcohol disorder and illicit drug use on TB treatment outcomes. Despite this negative interaction, a substantial proportion of unsuccessful treatment outcomes would be potentially preventable if both alcohol and drug exposures were eliminated from this population. This suggests that integrated public policies to prevent or reduce substance use in TB patients could have a meaningful impact on treatment outcomes and should be prioritized.

## Additional files

Additional file 1: **Table S1.** Profile study population with and without tuberculosis treatment outcome registration, São Paulo-state, Brazil, 2011–2015 (*n* = 79,075). (DOCX 23 kb)

Additional file 2: **Table S2.** Factors associated with unsuccessful tuberculosis treatment outcome with all unobserved treatment outcomes set to either successful (model 1) or unsuccessful (model 2), São Paulo-state, Brazil, 2011–2015 (n = 79,075). (DOCX 20 kb)

Additional file 3: **Figure S1.** Key messages of the association of alcohol disorder and illicit drug use on tuberculosis treatment outcome. São Paulo-state, Brazil, 2011–2015. (DOCX 329 kb)

## Abbreviations

CI: Confidence interval
DOT: Directly observed treatment
PAF: Population attributable fraction
RR: Relative risk
TB: Tuberculosis
TBWEB: Tuberculosis Cases Notification and Monitoring System
