# Supplementary material for: Impact of alcohol disorder and the use of illicit drugs on tuberculosis treatment outcomes: a retrospective cohort study
Source: Arch Public Health. 2018 Jul 12;76:45. doi: 10.1186/s13690-018-0287-z (PMC6042349; doi:10.1186/s13690-018-0287-z)
Supplement: Supplementary file 1 — Table S1. Profile study population with and without tuberculosis treatment outcome registration, São Paulo-state, Brazil, 2011–2015 (n = 79,075). (DOCX 23 kb) [file 13690_2018_287_MOESM1_ESM.docx]

**Table S1** Profile study population with and without tuberculosis treatment outcome registration, São Paulo-state, Brazil, 2011-2015 (n=79,075).

| Characteristics | Treatment outcomes registration | | | | Chi-square  P value^a^ |
| --- | --- | --- | --- | --- | --- |
|  | Yes | | No. | |  |
|  | No. | % | No. | % |  |
| Overall | 77,212 |  | 1,863 |  |  |
| Alcohol and drug | |  |  |  | 0.36 |
| Neither | 60,107 | 78.9 | 1470 | 77.8 |  |
| Only alcohol disorder | 7,487 | 8.5 | 158 | 9.7 |  |
| Only drug use | 5,199 | 6.7 | 124 | 6.7 |  |
| Alcohol disorder and drug use | 4,418 | 6.0 | 111 | 5.7 |  |
| No information | 1 | 0.0 | 0 | 0.0 |  |
| Sex |  |  |  |  | 0.34 |
| Female | 22,498 | 28.1 | 524 | 29.1 |  |
| Male | 54,714 | 71.9 | 1339 | 70.9 |  |
| Age (years) |  |  |  |  | 0.07 |
| 15-34 | 36,080 | 48.7 | 908 | 46.7 |  |
| 34-49 | 21,681 | 28.3 | 527 | 28.1 |  |
| 50 and more | 19,451 | 23.0 | 428 | 25.2 |  |
| Race |  |  |  |  | 0.22 |
| Non-black | 35,707 | 42.5 | 791 | 46.2 |  |
| Black | 33,135 | 41.9 | 781 | 42.9 |  |
| No information | 8,370 | 15.6 | 291 | 10.8 |  |
| HIV |  |  |  |  | **<0.001** |
| No | 69,973 | 86.8 | 1617 | 90.6 |  |
| Yes | 7,239 | 13.2 | 246 | 9.4 |  |
| Clinical form |  |  |  |  | **<0.001** |
| Pulmonary | 65,271 | 88.0 | 1640 | 84.5 |  |
| Extrapulmonary | 11,903 | 11.9 | 222 | 15.4 |  |
| No information | 38 | 0.1 | 1 | 0.0 |  |
| Prison |  |  |  |  | **<0.001** |
| No | 68,228 | 83.1 | 1548 | 88.4 |  |
| Yes | 8,984 | 16.9 | 315 | 11.6 |  |
| Homeless |  |  |  |  | **<0.001** |
| No | 75,218 | 92.5 | 1724 | 97.4 |  |
| Yes | 1,993 | 7.5 | 139 | 2.6 |  |
| Directly observed treatment | |  |  |  | **<0.001** |
| No | 16,791 | 24.9 | 463 | 21.7 |  |
| Yes | 54,940 | 56.4 | 1051 | 71.2 |  |
| No information | 5481 | 18.7 | 349 | 7.1 |  |

^a^Boldface indicates statistical significance (p<0.05).
