# Supplementary material for: Impact of alcohol disorder and the use of illicit drugs on tuberculosis treatment outcomes: a retrospective cohort study
Source: Arch Public Health. 2018 Jul 12;76:45. doi: 10.1186/s13690-018-0287-z (PMC6042349; doi:10.1186/s13690-018-0287-z)
Supplement: Supplementary file 2 — Table S2. Factors associated with unsuccessful tuberculosis treatment outcome with all unobserved treatment outcomes set to either successful (model 1) or unsuccessful (model 2), São Paulo-state, Brazil, 2011–2015 (n = 79,075). (DOCX 20 kb) [file 13690_2018_287_MOESM2_ESM.docx]

**Table S2** Factors associated with unsuccessful tuberculosis treatment outcome with all unobserved treatment outcomes set to either successful (model 1) or unsuccessful (model 2), São Paulo-state, Brazil, 2011-2015 (n= 79,075).

| Characteristics | Model 1 Adjusted RR  (95% CI)^a^ | Model 2 Adjusted RR  (95% CI)^a^ |
| --- | --- | --- |
|  |  |  |
| Overall |  |  |
| Alcohol and drug |  |  |
| Neither | 1 | 1 |
| Only alcohol disorder | **1.48 (1.4 - 1.56)** | **1.42 (1.35 - 1.49)** |
| Only drug use | **2.1 (1.98 - 2.21)** | **1.92 (1.82 - 2.02)** |
| Alcohol disorder and drug use | **2.1 (1.98 - 2.22)** | **1.94 (1.84 - 2.05)** |
| Sex |  |  |
| Female | 1 | 1 |
| Male | **1.27 (1.22 - 1.33)** | **1.22 (1.17 - 1.27)** |
| Age (years) |  |  |
| 15-34 | 1 | 1 |
| 34-49 | 0.99 (0.95 - 1.03) | 0.98 (0.94 - 1.02) |
| 50 and more | **1.23 (1.18 - 1.29)** | **1.19 (1.14 - 1.24)** |
| Race |  |  |
| Non-black | 1 | 1 |
| Black | **1.13 (1.09 - 1.17)** | **1.12 (1.08 - 1.15)** |
| HIV |  |  |
| No | 1 | 1 |
| Yes | **2.14 (2.05 - 2.24)** | **2.08 (1.99 - 2.16)** |
| Clinical form |  |  |
| Pulmonary | 1 | 1 |
| Extrapulmonary | **0.86 (0.82 - 0.91)** | **0.85 (0.81 - 0.89)** |
| Prison |  |  |
| No | 1 | 1 |
| Yes | **0.51 (0.46 - 0.55)** | **0.67 (0.62 - 0.72)** |
| Homeless |  |  |
| No | 1 | 1 |
| Yes | **1.96 (1.84 - 2.08)** | **1.98 (1.87 - 2.1)** |
| Directly observed treatment |  |  |
| No | 1 | 1 |
| Yes | **0.56 (0.54 - 0.58)** | **0.57 (0.55 - 0.59)** |

Abbreviations: RR, relative risk; 95% CI, 95% confidence interval.

^a^Boldface indicates statistical significance (p<0.05).
