# Supplementary figures and images for: Impact of alcohol disorder and the use of illicit drugs on tuberculosis treatment outcomes: a retrospective cohort study
Source: Arch Public Health. 2018 Jul 12;76:45. doi: 10.1186/s13690-018-0287-z (PMC6042349; doi:10.1186/s13690-018-0287-z)

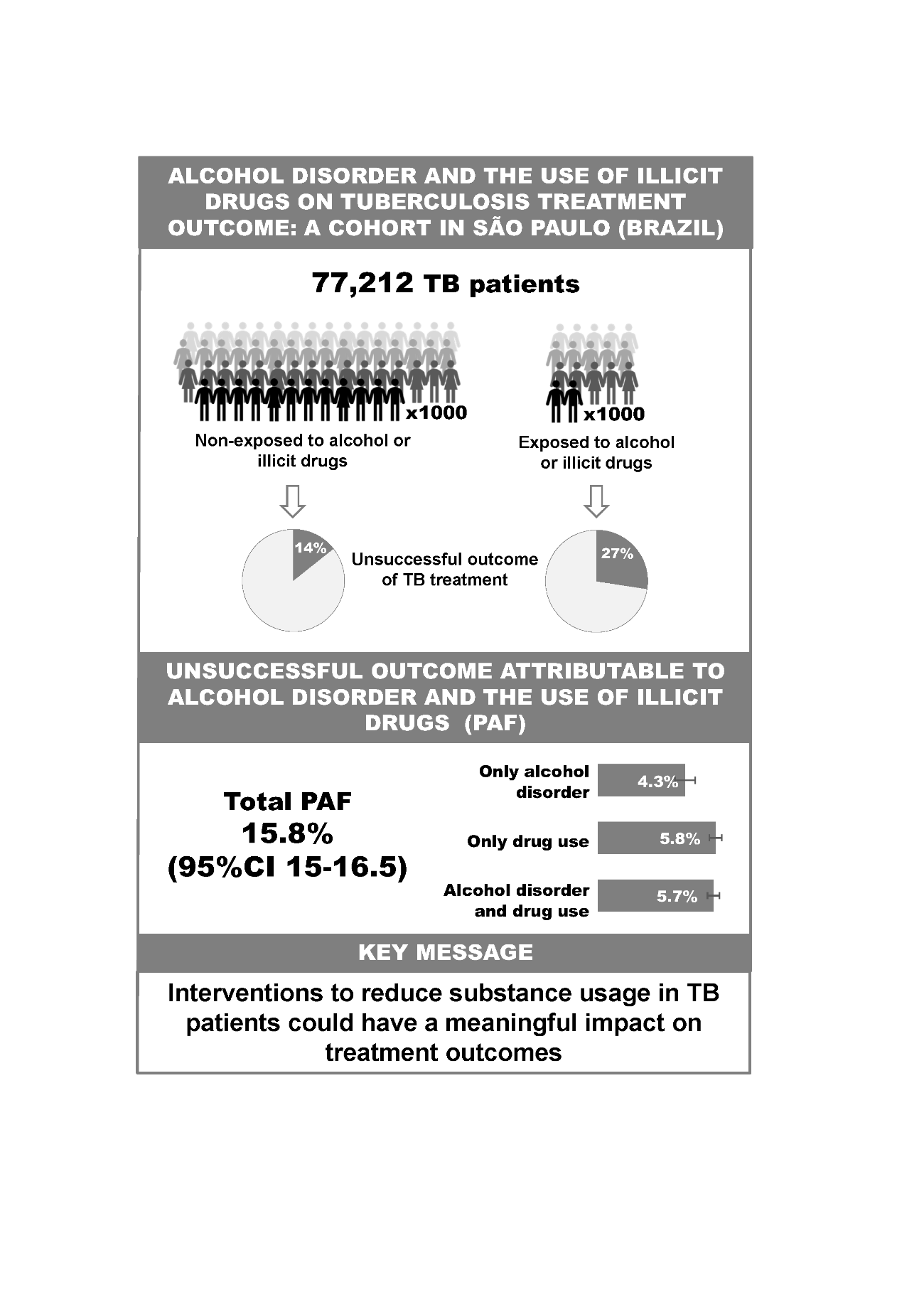

Supplement: Supplementary file 3 — Figure S1. Key messages of the association of alcohol disorder and illicit drug use on tuberculosis treatment outcome. São Paulo-state, Brazil, 2011–2015. (DOCX 329 kb) [file 13690_2018_287_MOESM3_ESM.docx]
